# Conditioned generalization of social anxiety effects based on positive and negative social evaluations

**DOI:** 10.1038/s41598-025-27857-2

**Published:** 2025-12-03

**Authors:** Huoyin Zhang, Qi Wu, Yukun Huang, Yujing Liu, Jingyuan Lin, Yi Lei

**Affiliations:** 1 Institute of Brain and Psychological Science, Sichuan Normal University, Chengdu, 610066 China; 2 School of Psychology, Shenzhen University, Shenzhen, 518060 China; 3 School of Education and Psychology, Southwest Minzu University, Chengdu, 610225 China; 4 School of psychology, Sichuan Normal University, Chengdu, 610068 China

**Keywords:** Social anxiety, Bivalent fear of evaluation, Conditional learning, Evaluations generalization, Online experiment, Psychology, Human behaviour

## Abstract

While negative social evaluations have been identified as a critical factor in social anxiety, the relationship between social anxiety (SA) levels and the generalization of positive and negative social evaluations remains unclear. To understand this relationship, 2 experiments were conducted. In Experiment 1, a total of 45 participants completed a laboratory-based conditioning and generalization task with neutral faces paired with positive or negative social evaluations. Using morphed versions of these faces as generalization stimuli, participants rated their expectancy of receiving social evaluations for each face. In Experiment 2, high SA and low SA groups (27 in each group) extended these findings by examining whether social anxiety levels modulated the generalization of social evaluations, with the addition of negative emotion ratings as a dependent measure. In Experiment 1, participants’ strong generalization of negative evaluations for stimuli similar to conditioned faces positively correlated with their SA levels. Experiment 2 also demonstrated a positive relation between SA and the generalization of negative evaluations for conditioned faces. Specifically, both high and low SA groups showed generalization in the form of gradual decreases in expectancy ratings as faces became less similar to the conditioned stimuli. However, the high SA group, compared to the low SA group, displayed enhanced negative emotional responses to faces paired with negative evaluations and their morphed variants, with this effect being strongest for faces most similar to the negative-paired faces (i.e., generalization). These results supported the cognitive processing model of social anxiety and highlight the importance of considering evaluation generalization in understanding and treating social anxiety.

**Supplementary Information:**

The online version contains supplementary material available at 10.1038/s41598-025-27857-2.

## Introduction

While much research has focused on individuals with social anxiety disorder (SAD), recent theoretical developments suggest that social anxiety exists along a severity continuum rather than as a discrete category^1,2^. Many individuals experience significant social anxiety symptoms without meeting full diagnostic criteria for SAD. Studying these subclinical manifestations can provide valuable insights into how social evaluation processes operate across the full spectrum of anxiety experiences. Therefore, this study examined social anxiety as a continuous trait variable, allowing us to investigate how varying levels of social anxiety related to the processing and generalization of social evaluations.

Fear conditioning paradigms provide a fundamental framework for understanding how fears are acquired and maintained. In these paradigms, a neutral stimulus (conditioned stimulus, CS) is repeatedly paired with an aversive event (unconditioned stimulus, US), resulting in the neutral stimulus acquiring the ability to elicit fear responses (conditioned response, CR) on its own. Traditionally, these paradigms employed basic aversive stimuli, such as electric shocks or loud noises as the US, paired with simple visual or auditory stimuli as the CS^3^. Through this associative learning process, the CS-US pairing leads to the formation of fear memories, where the previously neutral CS now independently triggers defensive responses similar to those originally elicited by the US^4,5^. Fear generalization, a process in which these conditioned fear responses (CRs) extend to stimuli sharing perceptual or conceptual similarities with the original CS but never directly paired with US, has been identified as a core mechanism in the development and maintenance of anxiety disorders^6,7^. Studies examining fear generalization in social anxiety have primarily used facial stimuli as the CS and negative social feedback or social rejection as the US. For instance, individuals with social anxiety show enhanced generalization of fear responses to faces sharing physical similarities with threat-conditioned faces^8^, and this overgeneralization tends to persist even when safety signals are present^8^. Additionally, social anxiety is associated with broader generalization gradients, meaning that conditioned fear responses extend to a wider range of stimuli that are less similar to the original CS^9^. However, these studies have largely focused on negative social evaluation as the US, neglecting the potential role of positive social feedback in fear learning and generalization processes.

In real-world social situations, individuals frequently encounter both positive and negative social evaluations, which often coexist^10^. While negative social evaluations have been traditionally viewed as the primary concern in social anxiety, recent theoretical developments in the bivalent fear of evaluation (BFOE) model emphasize that both fear of negative evaluation (FNE) and fear of positive evaluation (FPE) contribute to the maintenance of social anxiety disorder^11^. These fears operate through distinct pathways; FNE primarily relates to immediate threat detection and avoidance, whereas FPE involves more complex cognitive processes related to social status concerns and fear of increased social expectations^12^. Research has found that both types of evaluation fears show significant positive correlations with social anxiety^13^ and can predict its development^14^. However, the cognitive mechanisms underlying how these evaluation fears influence information processing and generalize across different social contexts remain unclear, which is the focus of the current study.

To systematically investigate these research questions, we conducted both laboratory and web-based experiments. Recent advances in Web-based research methodologies have demonstrated their reliability and validity in psychological research. Studies comparing Web and laboratory (Lab) data have shown comparable data quality across various psychological effects^15^. While Web-based experiments may have slightly increased response latencies compared to Lab versions, these delays are typically constant within participants and, thus, do not affect the measurement of experimental effects that rely on within-subject comparisons^16^. Web-based methods offer several advantages, including enhanced experimental control, automated data collection, and improved internal validity. These methods allow for precise environmental control, reduced interference, and efficient collection of large-scale data^17,18^. Combining Web and Lab approaches can provide more comprehensive results^19–21^.

Our study addresses this gap and extends previous research in three important ways. First, drawing from the Bivalent Fear of Evaluation (BFOE) model, we examine processing biases for both positive and negative evaluations. This theoretical advance suggests that socially anxious individuals show unique processing characteristics not only for negative feedback but also for positive social evaluations^10,22^. Second, while extensive research has examined fear conditioning in social anxiety, recent meta-analyses have highlighted the instability of extinction effects^23^. We extend beyond basic acquisition and extinction processes to investigate generalization mechanisms, which involve more sophisticated cognitive operations, including similarity judgment and threat assessment of novel stimuli. This approach allows us to better understand how socially anxious individuals process and generalize social threats across different contexts. Finally, our study employs both laboratory and online experimental paradigms, offering complementary methodological strengths. While laboratory settings provide precise environmental control necessary for fear conditioning research^24^, online experiments enhance ecological validity and allow for more efficient data collection from diverse samples^17,18^. This dual-method approach provides a more comprehensive understanding of fear generalization processes in social anxiety.

**Fig. 1:**
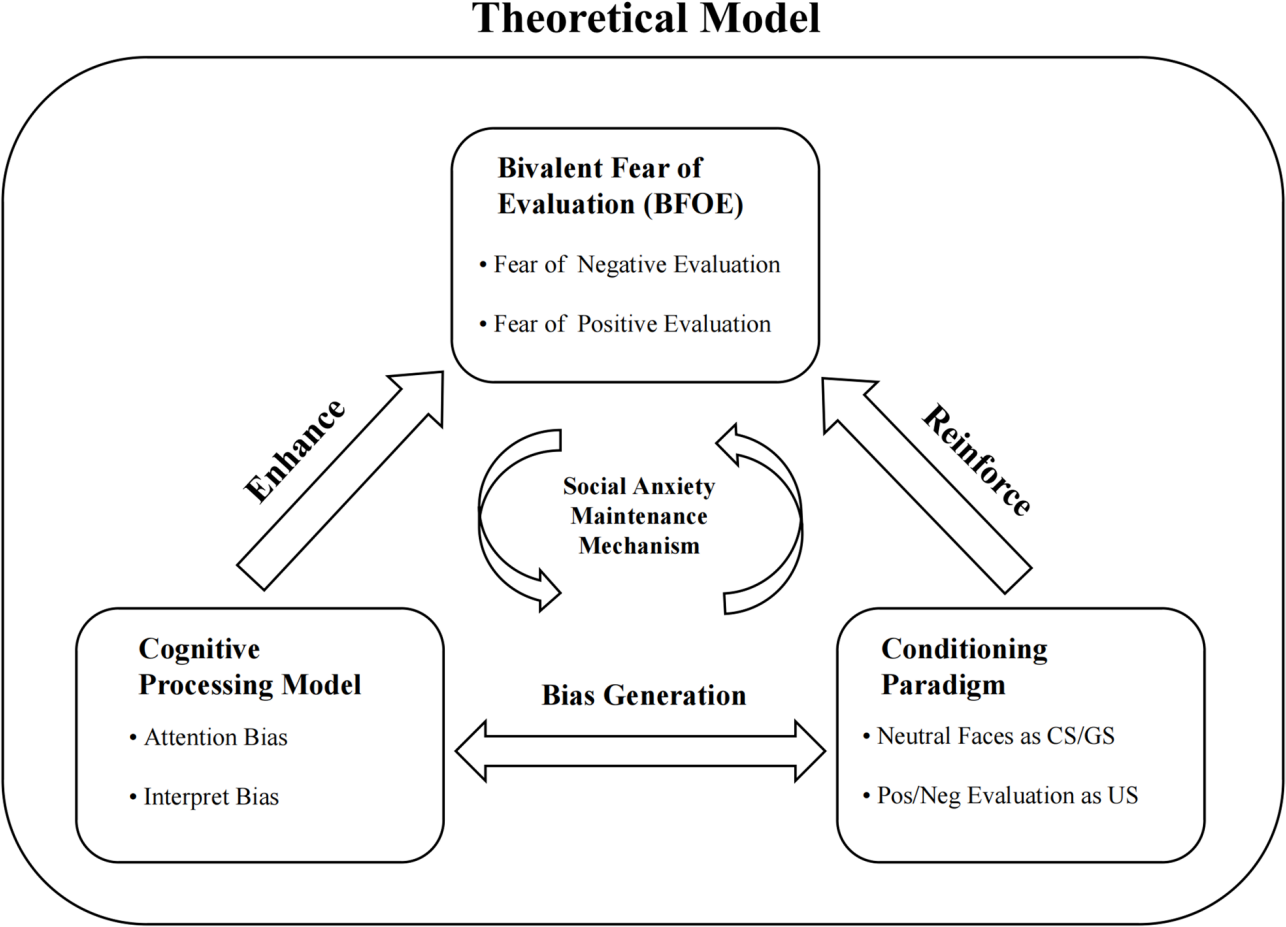
Evaluative Generalization in Social Anxiety: An Integrated Theoretical Model. This theoretical model proposes Bivalent Fear of Evaluation (BFOE) as a core construct, encompassing both negative and positive evaluation fears. The model integrates three primary components and their hypothesized interactions: The cognitive processing component, with its attention and interpretation biases, potentially enhances BFOE; The conditioning component, where neutral faces serve as conditioned/generalized stimuli (CS/GS) and evaluations as unconditioned stimuli (US), may reinforce BFOE. Between these components, a bidirectional “bias generation” mechanism suggests that cognitive biases influence conditioning processes, while learning experiences modulate cognitive biases. The proposed maintenance mechanism at the model’s center indicates how BFOE, cognitive processing, and conditioning might maintain social anxiety symptoms through their reciprocal interactions.

While significant progress has been made in understanding social anxiety and evaluation processing, several key questions emerging from previous research remain to be addressed. This study aims to explore the cognitive processing abnormalities in individuals with SA and their impact on evaluation generalization. To bridge this gap in understanding the cognitive mechanisms underlying BFOE, we propose an integrated theoretical model (Fig. 1) that positions Bivalent Fear of Evaluation (BFOE) as a core construct, with three primary components: cognitive processing, conditioning, and their bidirectional interactions through a “bias generation” mechanism. Drawing from this theoretical model, we position BFOE as a core construct that influences both cognitive processing and conditioning mechanisms. Building on the principles of fear conditioning, we introduce positive and negative social evaluations as the US and pair them with neutral faces as CS. This approach allows us to assess CR and explore how fear generalization occurs when participants are exposed to novel but similar faces. The innovation of this study lies in testing our theoretical model’s propositions about how cognitive biases and conditioning processes interact bidirectionally in the context of social anxiety. Both laboratory and web-based experiments were conducted to provide comprehensive validation of these theoretical mechanisms.

Based on our theoretical model and previous research, we proposed the following hypotheses: (1) Participants were expected to exhibit conditional generalization effects, as reflected in high US expectancy ratings for novel stimuli that closely resemble the original conditioned stimuli (CS) paired with both positive and negative social evaluations, consistent with our model’s BFOE component; (2) The conditional generalization effect was expected to be stronger for negative social evaluations than for positive social evaluations, as reflected in higher US expectancy ratings for negative evaluation-associated stimuli, aligning with our model’s predictions about the distinct mechanisms of FNE and FPE; (3) Participants with high levels of social anxiety (SA) were expected to show significantly higher US expectancy ratings for novel stimuli associated with negative social evaluations compared to participants with low levels of SA, reflecting greater fear generalization and the enhanced bidirectional relationships between cognitive biases and conditioning processes proposed in our model. Verifying these hypotheses was expected to help validate our theoretical model while clarifying the cognitive processing mechanisms in SA, particularly how the interaction between cognitive biases and conditioning processes maintains social anxiety symptoms.

## Experiment 1: the relationship between generalization of positive and negative social evaluations and social anxiety

### Materials and methods

#### Participants

To further ensure the adequacy of the sample size and the statistical power of the study, an a priori power analysis was conducted using G*Power 3.1. For the 2 (evaluation type: positive, negative) × 6 (stimulus type: CS−, GS1, GS2, GS3, GS4, CS+) repeated measures ANOVA design, with an anticipated effect size f = 0.25 (medium effect), α = 0.05, power = 0.80, the minimum required sample size was 13 participants. The sample size was determined based on prior studies using similar conditioning paradigms^3,8^. Among the 56 participants who underwent eligibility screening, 7 were excluded during the experimental phase: 2 due to technical issues with equipment and 5 did not participate in the experiment. A total of 49 participants completed the experiment. During data validation, 4 participants were excluded due to incomplete data. No participants were excluded during the analysis phase. The final sample included 45 participants (34 females, mean age = 20.51 ± 1.80) whose data were included in the data analysis (see Table 1 and Supplementary Fig. 1). Informed consent was obtained from each participant prior to their involvement in the study. All participants were right-handed, self-reported no mental illness, and reported normal or corrected vision. Participants were informed they could withdraw from the experiment at any time if discomfort arose. This study was approved by the Ethics Committee of the Institute of Brain and Psychological Sciences at Sichuan Normal University (SCNU-211120) and was conducted in accordance with the latest revision of the Declaration of Helsinki.

**Table 1:** Descriptive statistics of participant demographics and questionnaires in experiment 1. (*M* ± *SD*).

Sex	Number	Age	LSAS	LSAS-Fear/Anxiety	LSAS-Avoidance
Male	11	19.91 ± 1.45	53.09 ± 18.50	26.36 ± 12.70	26.73 ± 7.49
Female	34	20.71 ± 1.90	52.76 ± 17.10	26.35 ± 11.83	26.41 ± 8.52
Total	45	20.51 ± 1.80	52.84 ± 17.23	26.36 ± 11.90	26.49 ± 8.20

*LSAS* Liebowitz Social Anxiety Scale, *M* mean, *SD* standard deviation.

#### Materials

##### Measurement tool

The main measure used in this experiment was the Chinese version of the Liebowitz Social Anxiety Scale (LSAS)^25,26^, which consists of 24 items covering social situations (11 items) and performance situations (13 items), assessing fear, anxiety, and avoidance. The scale is divided into four subscales: performance fear, performance avoidance, social fear, and social avoidance. The assessment period included the past 3 months “Fear or anxiety” refers to subjective experience and is rated on a scale ranging from 0 to 3, representing none, mild, moderate, and severe. “Avoidance” refers to objective avoidance frequency and is rated on a scale ranging from 0 (*never*) to 3 (*almost always*), with a 1/3 increase in frequency for each level. Additionally, item 25 of the scale lists the three most feared situations from the aforementioned items and is not included in the total score. In this experiment, the Cronbach’s α coefficient for the scale was 0.93, with Cronbach’s α coefficients of 0.94 and 0.84 for the fear/anxiety subscale and the avoidance subscale, respectively.

##### Experimental stimuli

The conditioned stimuli (CS) were neutral facial images of three female individuals from the NimStim face expression database^27^. The unconditioned stimuli (US) consisted of 12 evaluative statements (6 positive, 6 negative). Positive US included statements like “you are really funny”, whereas negative US included “you are really selfish”. The evaluative statements were all selected from the Chinese Personality Adjectives Pool^28^. The conditioned stimuli (CS) appeared in combination with the US-positive (CS + positive) and the US-negative (CS + negative), respectively, to form a conditioned response; whereas unpaired stimuli were labeled CS−. The generalization stimuli (GS) were female facial images that gradually morphed from CS + to CS−. Using FaceMorpher software (version 2.5 Lite, Luxand Inc. Alexandria, VA), morphing was performed at a degree of 20%, resulting in four levels of morphed faces (GS1, GS2, GS3, GS4), with 4 positive and 4 negative faces. Among them, GS4 was the most similar to the CS+, whereas GS1 was the most similar to the CS−^29^.

#### Procedure

The experiment consisted of two sessions. In the first session, participants were asked to provide a passport-sized color photograph and participated in an online photo evaluation task which demanded approximately 5 min to complete. During this task, participants were presented with a series of facial photographs to evaluate, and were told that their own photograph would be evaluated by other participants in return. This setup was designed to create an interactive social evaluation context (in reality, there were no other participants; the photos being evaluated were pre-selected from the face database, and participants’ photos were not actually shared or evaluated by others). After completing this online evaluation session, participants were invited to the laboratory for the second session. The second session was conducted in a sound-attenuated laboratory and programmed using E-Prime 3.0 (Psychological Software Tools, Inc., Pittsburgh, PA). Visual stimuli were presented on a 23-inch LCD monitor (1920 × 1080 pixels, 60 Hz refresh rate). Participants were seated approximately 60 cm from the screen. The visual stimuli (neutral faces) were presented with visual angles of 6.16° × 7.82°.

Upon arrival at the laboratory, participants first provided written informed consent and completed a demographic questionnaire (including age and gender). They, then, completed the LSAS to assess baseline social anxiety levels. After these preliminary procedures, the experiment consisted of three phases: Habituation phase, Acquisition phase and Generalization phase. In the Habituation phase, the CS + positive, CS + negative and CS − were presented 3 times in pseudo-randomized order without any US, to familiarize participants with the procedure. In the Acquisition phase, the CS + positive, CS + negative and CS − were presented in 24 trials (8 for each), with CS + positive paired to positive US and CS + negative to negative US at a 75% reinforcement rate. The CS − was not paired with any US. In the Generalization phase, four blocks were presented, with each block presenting 2 trials of CS + positive, CS + negative, and CS − stimuli, as well as 2 trials of each type of generalization stimulus (GS), resulting in a total of 22 trials. Randomly selected CS + positive or CS + negative were reinforced once per block. Participants were allowed to take self-paced breaks between blocks to minimize fatigue.

**Fig. 2:**
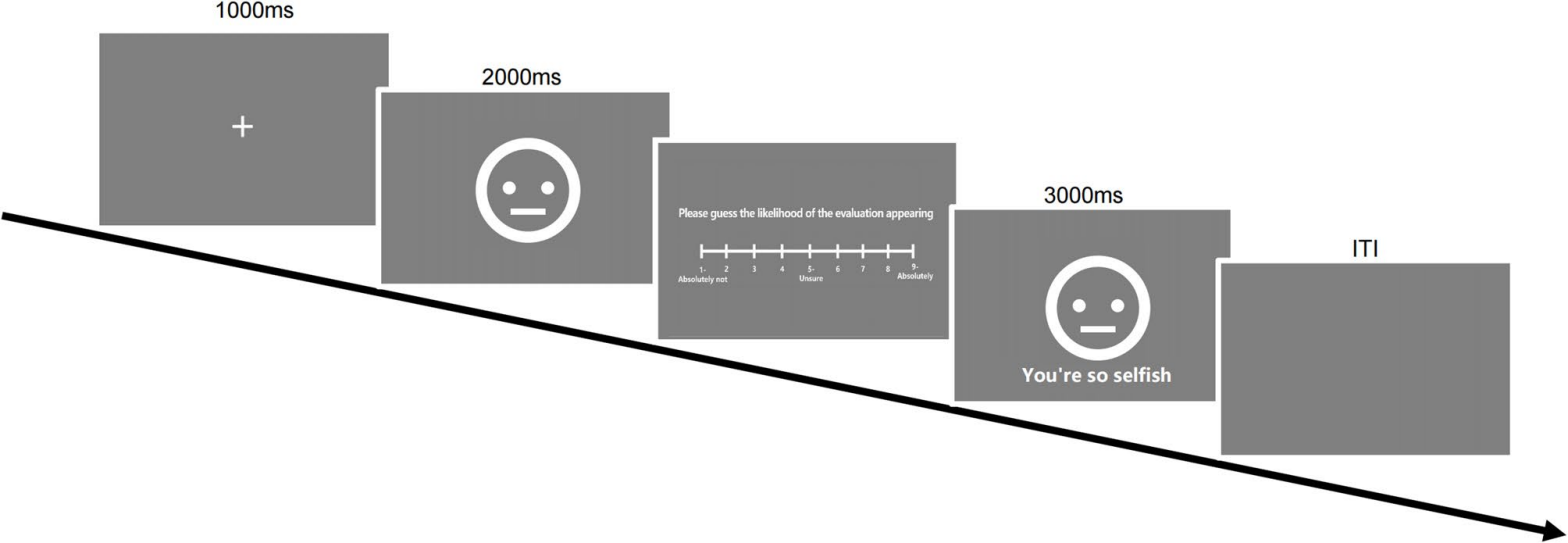
Across both experiments, each trial followed a similar procedure. A 1000 ms fixation (+) was followed by a 2000 ms CS or GS. Participants then rated their US expectancy in response to the prompt, “Please guess the likelihood of receiving an evaluation.” Subsequently, a face, with or without a social evaluation, appeared for 3000 ms. The experiments differed in two primary ways. Experiment 1 featured a longer inter-trial interval (ITI) of 9000–12,000 ms, compared to 1500–5500 ms in Experiment 2. Furthermore, Experiment 2 uniquely required participants to provide retrospective negative emotion ratings for all stimuli at the conclusion of the acquisition and generalization phases.

Each trial began with a 1000 ms fixation “+”, followed by a 2000 ms CS or GS presentation. Subsequently, the probe text “Please guess the likelihood of receiving an evaluation” appeared at the center of the screen, requiring participants to provide US expectancy ratings on a 9-point scale (1–9). Participants then provided US expectancy ratings, followed by a 3000 ms presentation of a face with or without an evaluation. The inter-trial interval (ITI) was jittered between 9000 and 12,000 ms to reduce temporal predictability. Responses were collected using a standard keyboard. The second session demanded 60 to 70 min. All participant responses and timing data were automatically logged for subsequent analysis. (Fig. 2)

#### Subjective measures (US expectancy ratings)

After each CS or GS was shown for 2000 ms, participants rated the likelihood of receiving an evaluation on a 9-point Likert-type scale. Ratings were made using the right hand on a keyboard. A rating of 1 indicated no evaluation at all, whereas a rating of 9 indicated an absolute certainty of receiving an evaluation, with high numbers representing a strong likelihood of the US occurring.

#### Data analysis

Statistical analyses were conducted using SPSS 27.0 (IBM Corporation, Armonk, NY, USA), with the dependent variable being the US expectancy ratings provided by the participants. For the acquisition phase, a single-factor repeated-measures ANOVA was conducted on CS + positive, CS−, and CS + negative stimuli. For the generalization phase, a 2 (evaluation type: positive, negative) × 6 (stimulus type: CS−, GS1, GS2, GS3, GS4, CS+) two-factor repeated measures ANOVA was performed. Linear slope fitting was applied to the generalization data using MATLAB R2016a. with low slopes indicating great generalization^30^. Paired-sample *t*-tests were conducted on the fitted slopes for positive and negative evaluations using SPSS 27.0. Finally, Pearson correlation analyses were conducted between LSAS scores (total, fear/anxiety, avoidance) and generalization slopes. Greenhouse-Geisser corrections were applied where necessary, and Bonferroni corrections were used for pairwise comparisons. Two-tailed p-values determined statistical significance, and η_p_² and Cohen’s *d* were used to report effect sizes.

### Results

#### Acquisition phase

**Fig. 3:**
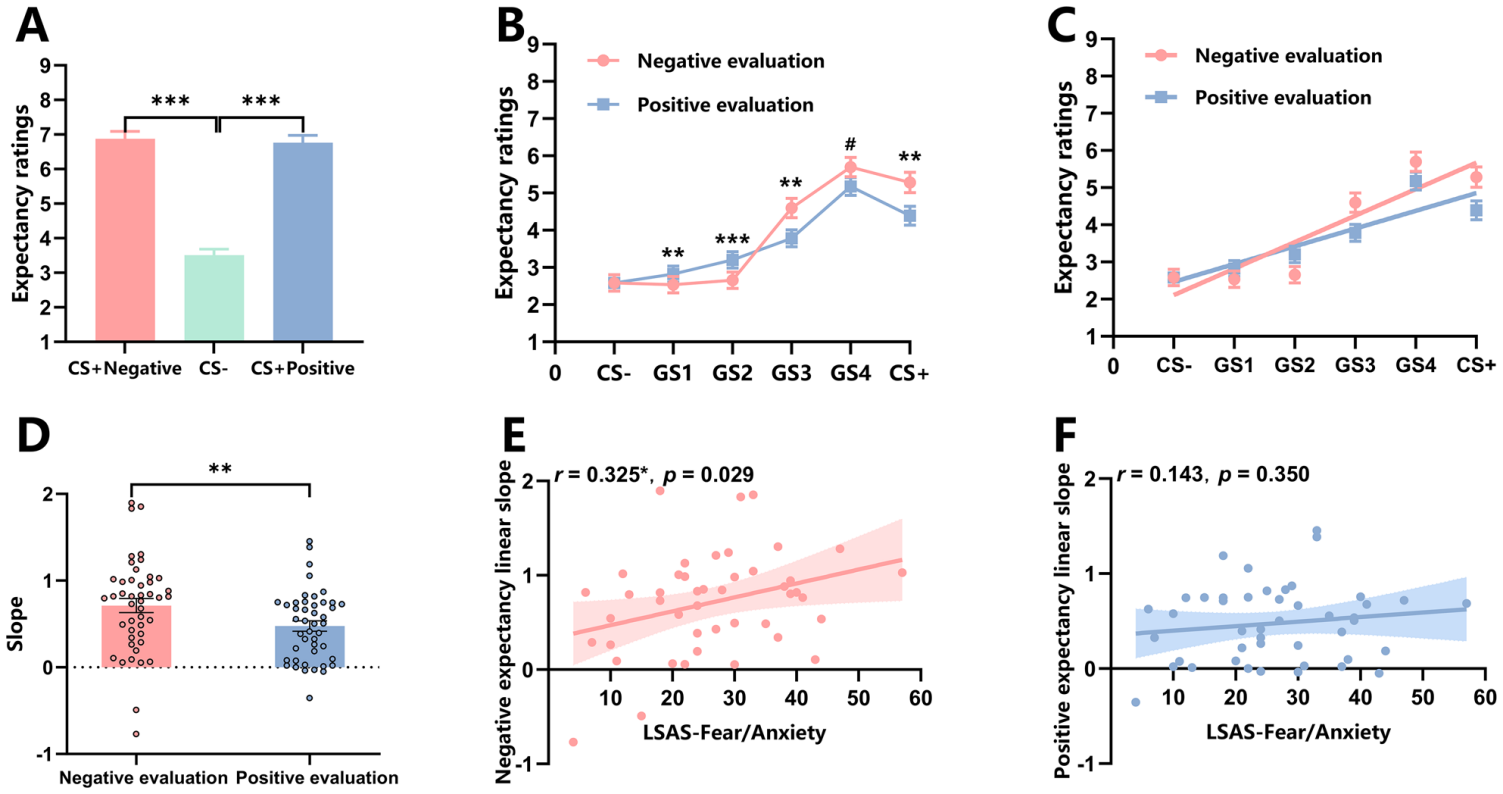
Results of all data analyses in Experiment 1 (*M* ± *SE*). (**A**) Results of the acquisition phase; (**B**) Results of the generalization phase; (**C**) Results of linear slope fitting in the generalization phase; (**D**) Results of slopes in the generalization phase; (**E**,**F**) Correlation analysis results between LSAS-Fear/Anxiety total score and linear slopes of US expectancy ratings. ***: *p* < 0.001, **: *p* < 0.01, *: *p* < 0.05, # indicates *p* = 0.081.

The main effect of stimulus type (CS + vs. CS−) was significant, *F*(2, 88) = 81.38, *p* < 0.001, η_p_^2^ = 0.65. Post-hoc tests showed that participants’ US expectancy ratings were higher for the CS + negative (*M* = 6.87, *SD* = 1.43) than the CS− (*M* = 3.50, *SD* = 1.20), *t*(44) = 10.24, *p* < 0.001, Cohen’s *d* = 1.53. The US expectancy ratings were higher for the CS + positive *(M* = 6.77, *SD* = 1.42) than the CS− (*M* = 3.50, *SD* = 1.20), *t*(44) = 10.68, *p* < 0.001, Cohen’s *d* = 1.59. However, no significant difference was found in US expectancy ratings between the CS + negative and CS + positive, *t*(44) = 0.41, *p* = 0.68, Cohen’s *d* = 0.06 (Fig. 3A). These results indicated that participants successfully acquired the association between CS and US, and the learning of positive and negative social evaluations was equivalent.

#### Generalization phase

##### Raw data of expectancy ratings

The main effect of stimulus type was significant, *F*(1.68, 73.89) = 72.91, *p* < 0.001, η_p_^2^ = 0.62. Post-hoc pairwise comparisons showed that participants’ US expectancy ratings was higher for the CS+ (*M* = 4.84, *SD* = 1.82), GS4 (*M* = 5.44, *SD* = 1.68), GS3 (*M* = 4.19, *SD* = 1.69), and GS2 (*M* = 2.93, *SD* = 1.49) than the CS− (*M* = 2.58, *SD* = 1.47, *ps* < 0.001, *p* = 0.001). No significant difference in US expectancy ratings were found between the GS1 and CS− (*p* = 1.00), indicating that individuals’ generalization of evaluations reached the GS2 level. The main effect of evaluation type was marginally significant, *F*(1, 44) = 3.05, *p* = 0.088, η_p_^2^ = 0.65. Post-hoc pairwise comparisons showed that participants’ US expectancy ratings were significantly higher for negative evaluations (*M* = 3.89, *SD* = 2.11) than positive evaluations (*M* = 3.66, *SD* = 1.78), suggesting that individuals are more sensitive to negative evaluations.

The interaction effect between stimulus type and evaluation type was significant, *F*(2.00, 87.95) = 12.83, *p* < 0.001, η_p_^2^ = 0.23. A simple main effects analysis revealed that participants’ US expectancy ratings were higher for negative evaluations than positive evaluations at the levels of the CS+, GS4, and GS3 (CS + negative (*M* = 5.28, *SD* = 1.85) vs. CS + positive (*M* = 4.39, *SD* = 1.70), *p* = 0.002; GS4 negative (*M* = 5.70, *SD* = 1.74) vs. GS4 positive (*M* = 5.18, *SD* = 1.61), *p* = 0.081; GS3 negative (*M* = 4.60, *SD* = 1.77) vs. GS3 positive (*M* = 3.79, *SD* = 1.53), *p* = 0.003). However, at the levels of GS2 and GS1, participants’ US expectancy ratings were higher for positive evaluations than negative evaluations (GS2 positive (*M* = 3.20, *SD* = 1.48) vs. GS2 negative (*M* = 2.66, *SD* = 1.46), *p* < 0.001; GS1 positive (*M* = 2.82, *SD* = 1.45) vs. GS1 negative (*M* = 2.53, *SD* = 1.50), *p* = 0.002, (Fig. 3B and C). Under negative evaluations, participants’ US expectancy ratings were higher for CS+ (*M* = 5.28, *SD* = 1.85), GS4 (*M* = 5.70, *SD* = 1.74), and GS3 (*M* = 4.60, *SD* = 1.77) than CS− (*M* = 2.58, *SD* = 1.47, *ps* < 0.001), with no significant differences between the GS1, GS2, and CS− (*ps* = 1.00). Under positive evaluations, however the US expectancy ratings were significantly higher for the CS+ (*M* = 4.39, *SD* = 1.70), GS4 (*M* = 5.18, *SD* = 1.61), GS3 (*M* = 3.79, *SD* = 1.53), GS2 (*M* = 3.20, *SD* = 1.48), and GS1 (*M* = 2.82, *SD* = 1.45) than for the CS− (*M* = 2.58, *SD* = 1.47) (*ps* < 0.039).

##### Slopes of expectancy ratings

The paired-sample *t*-test results showed that the linear slope of US expectancy ratings were higher for negative evaluations (*M* = 0.71, *SD* = 0.54) than positive evaluations (*M* = 0.48, *SD* = 0.40), *t*(44) = 3.56, *p* = 0.001, Cohen’s *d* = 0.53 (Fig. 3D).

#### Correlation analysis

The results of the Pearson correlation analysis are presented in Table 2. The LSAS-Fear/Anxiety total score was significantly positively correlated with the linear slope of US expectancy ratings for negative evaluations (*r* = 0.325, *p* = 0.029) (Fig. 3E,F). All other correlations were not significant (*ps* > 0.05).

**Table 2:** Correlation analysis results between LSAS and its subscales and linear slopes of expectancy ratings in experiment 1.

	Linear slope of negative evaluation expectancy ratings	Linear slope of positive evaluation expectancy ratings
LSAS	0.212	0.053
LSAS-Fear/Anxiety	0.325^*^	0.143
LSAS-Avoidance	−0.026	−0.095

*p < 0.05.

### Discussion

The results showed that participants successfully acquired the CS-US association, forming the basis for generalization. In the generalization phase, participants demonstrated higher expectancies of negative evaluations than positive evaluations for stimuli similar to CS+, indicating a generalization bias towards negative evaluations. Additionally, the fear component of social anxiety was positively correlated with this bias, suggesting that high levels of social anxiety were related to high sensitivity to negative evaluations as well as a high degree of generalization.

## Experiment 2: the effect of social anxiety on the generalization of positive and negative social evaluations

While Experiment 1 provided initial evidence for differential generalization patterns, several limitations needed to be addressed. First, the lab sample primarily consisted of university students with an uneven gender distribution, potentially limiting the generalizability of our findings. Second, the experiment focused solely on US expectancy ratings, which might not fully capture the emotional aspects of fear generalization. To address these limitations, we conducted Experiment 2 using a web paradigm that enabled recruitment of a more diverse and gender-balanced sample. The larger sample size also allowed for meaningful comparisons between high and low social anxiety groups. Furthermore, we expanded our dependent measures to include negative emotional ratings alongside US expectancy ratings, providing a more comprehensive assessment of both cognitive and emotional aspects of fear generalization in social anxiety.

### Materials and methods

#### Participants

To ensure the adequacy of the sample size and statistical power, an a priori power analysis was conducted using G*Power 3.1. For the 2 (group: high SA, low SA) × 2 (evaluation type: positive, negative) × 6 (stimulus type: CS−, GS1, GS2, GS3, GS4, CS+) mixed repeated measures ANOVA design, with an anticipated effect size f = 0.25 (medium effect), α = 0.05, power = 0.80, the analysis suggested a minimum sample size of 28 participants. The sample size was determined based on prior studies using similar paradigms^31^ For the series of studies, a participant pool was established through the Naodao Platform^32^ and social media, collecting 1,445 valid responses (78% valid response rate) from a total of 1,358 social anxiety questionnaires distributed. For this specific sub-study, to ensure distinct group differences in social anxiety levels, we specifically recruited participants from the upper and lower 20% of the LSAS score distribution. During the recruitment phase, 87 participants underwent eligibility assessment. During the experimental phase, 26 participants were excluded: 17 participants who did not participate in the experiment and 9 participants who were eliminated due to experimental system issues. A total of 61 participants completed the experiment. During completed the experiment, 2 participants were excluded due to incomplete data. In the subsequent analysis phase, 5 additional participants were excluded for failing attention checks. The final sample included 54 participants, who met al.l technical requirements for online participation (desktop or laptop computer with stable internet connection, quiet environment). The participants were divided into high SA group (27 participants; 13 females, mean age = 21.56 ± 1.99) and low SA group (27 participants; 14 females, mean age = 22.04 ± 3.25)^33^. All participants were right-handed, reported normal or corrected vision, and no psychiatric history. Informed consent was obtained from all participants prior to their involvement in the study^34^. This study was approved by the Ethics Committee of the Institute of Brain and Psychological Sciences at Sichuan Normal University (SCNU-211120) and was conducted in accordance with the latest revision of the Declaration of Helsinki.(see Table 3 and Supplementary Fig. 2).

**Table 3:** Demographic variables and scale statistical results of high SA and low SA participants in experiment 2 (*M* ± *SD*).

Variable	High SA group (*n* = 27)	Low SA group (*n* = 27)	Statistical indices			
			t_52_/ χ^2^	*p*	Cohen’s d	95% CI
Sex (Male/Female)	14/13	13/14	0.07	0.785		
Age	21.56 ± 1.99	22.04 ± 3.25	−0.66	0.511	0.18	[− 0.71, 0.36]
LSAS	81.41 ± 15.21	19.44 ± 8.45	18.45***	< 0.001	5.02	[3.91, 6.11]
LSAS-Fear/Anxiety	43.70 ± 8.88	9.44 ± 5.60	16.96***	< 0.001	4.61	[3.58, 5.64]
LSAS-Avoidance	37.77 ± 10.00	10.00 ± 3.89	13.42***	< 0.001	3.65	[2.77, 4.53]

LSAS: Liebowitz Social Anxiety Scale; *M*: mean; *SD*: standard deviation; Sex was assessed using a chi-square test, while age, LSAS, and its subscales were analyzed using independent samples t-tests; n: sample size; ***: *p* < 0.001; Cohen’s *d*: effect size for t-tests, 0.2 ≤ Cohen’s *d* < 0.5 indicates small effect size, 0.5 ≤ Cohen’s *d* < 0.8 indicates medium effect size, Cohen’s *d* ≥ 0.8 indicates large effect size.

#### Materials

All materials used in Experiment 2 were identical to the ones used in Experiment 1, including: (1) The Chinese version of the LSAS for assessing social anxiety levels (Cronbach’s α = 0.98, with fear/anxiety subscale = 0.97 and avoidance subscale = 0.96 in the current sample). (2) The same set of conditioned stimuli (CS), unconditioned stimuli (US), and generalization stimuli (GS) from the NimStim face expression database.

#### Procedure

The experiment was conducted entirely online in two phases: screening phase and experimental phase. Based on the LSAS scores collected during the initial screening, participants were divided into high and low social anxiety groups. The primary aim of conducting Experiment 2 online was to extend the ecological validity of Experiment 1’s findings. The secondary aim was to select participants who were high and low in social anxiety and to balance gender across those groups. The multi-person online evaluation task preceded the formal experiment. All participants provided specific consent for this procedure. Similar to Experiment 1, participants were asked to provide a passport-sized color photograph and participated in an online photo evaluation task which took approximately 5 min.

During this task, participants evaluated a series of facial photographs and were informed that their photograph would be evaluated by other individuals (in reality, there were no other participants; all photos were pre-selected from the face database). Prior to the experiment, participants were required to use a desktop or laptop computer (not tablets or mobile phones) with a stable internet connection. They were instructed to complete the experiment in a quiet environment with minimal distractions. Experiment 2 was conducted online through the NaoDao web platform. Visual stimuli were presented on participants’ own computer monitors, and they were instructed to sit approximately 60 cm from their screens. Similar to Experiment 1, the experiment consisted of three phases: Habituation, Acquisition, and Generalization. In the Habituation phase, 3 presentations were made for the CS + positive, CS + negative, and the CS- stimuli without the US. In the Acquisition phase, 24 trials (8 each for CS + positive, CS + negative and CS−) were completed with a 75% reinforcement rate. In the Generalization phase, 4 blocks were conducted with 22 trials.

Experiment 2 differed from Experiment 1 in the five following ways. After completing the acquisition and generalization phases, participants in Experiment 2 underwent an attention check, specifically by determining whether they could correctly identify CS + positive, CS + negative, and CS−. Participants who failed the check were excluded. In addition, the CS + positive and CS + negative were balanced between groups and individuals (within the high SA group, the CS + positive and CS + negative were balanced across the participants, and the same balancing procedure applied to the low SA group). Furthermore, all participant responses were made using computer keyboard, including both US expectancy and negative emotion ratings. The formal experimental procedure was programmed using PsychoPy v.2021.2.3 on the NaoDao web platform (https://www.psychopy.org/). PsychoPy was chosen for its precise timing capabilities and flexibility in online experimental settings. Finally, the inter-trial interval ranged from 1500ms to 5500ms in Experiment 2.

To prevent fatigue during the online experiment, participants were allowed to take short breaks between experimental phases. A progress bar was displayed to help participants track their progression through the experiment. Following the completion of the acquisition and generalization phases, participants provided retrospective negative emotion ratings for the stimuli. The entire experimental session lasted approximately 60–70 min, consistent with Experiment 1. All participant responses and timing data were automatically logged through the online platform for subsequent analysis. (Fig. 2)

#### Quality control measures

To ensure data quality in our Web-based experiment, we implemented several control measures. First, experimenters remained available throughout the online sessions to provide real-time support and address any technical or procedural questions from participants. Second, we included attention checks and exclusion criteria for technical issues. These monitoring and control measures helped maintain experimental rigor and address common concerns about online data quality, such as participant attention and technical variability^35^.

#### Subjective measures

First, US expectancy ratings were collected in the same manner as in Experiment 1. During each trial, participants rated “How likely do you think you will receive an evaluation?” on a 9-point scale, where 1 indicated “definitely no evaluation” and 9 indicated “definitely evaluation”.

Second, negative emotional ratings were collected retrospectively at the end of the acquisition and generalization phases. Specifically, after completing each phase, participants provided a single, summary rating for their subjective negative emotional experience toward each type of stimulus (CS and GS) based on their overall experience throughout that phase. Participants were asked to rate their subjective negative emotional experiences for the CS and GS on a scale ranging from 1 to 9, with 1 representing “not negative at all” and 9 representing “extremely negative.” High values indicated strong negative emotions experienced by the participants.

#### Data analysis

Data were analyzed using SPSS 27.0. The dependent variables were US expectancy and negative emotion ratings. For the acquisition phase, a 2 (group: high SA, low SA) × 3 (stimulus type: CS + positive, CS + negative, CS−) mixed-factorial design and an ANOVA were used to evaluate the measures. For the generalization phase, a 2 (group: high SA, low SA) × 2 (evaluation type: positive, negative) × 6 (stimulus type: CS−, GS1, GS2, GS3, GS4, CS+) mixed-factorial design and an ANOVA were conducted to evaluate the measures. The MATLAB R2016a program was used for linear slope fitting on the expectancy and emotion ratings. A 2 (group: high SA, low SA) × 2 (evaluation type: positive, negative) mixed-design ANOVA was conducted on the slopes. Greenhouse-Geisser corrections were applied when necessary, and Bonferroni adjustments were used for pairwise comparisons. Two-tailed p-values determined statistical significance, and η_p_² and Cohen’s *d* were used to report effect sizes. 

### Results

#### Acquisition phase

**Fig. 4:**
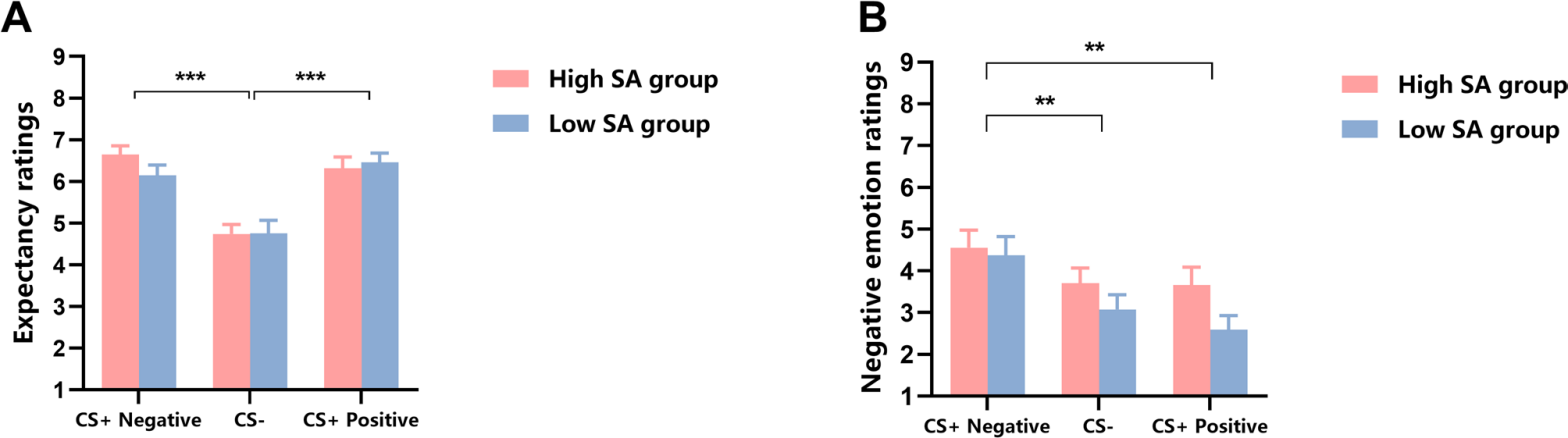
US expectancy and negative emotion ratings results for high and low SA groups across the acquisition phase in Experiment 2 (*M* ± *SE*). (**A**) show the US expectancy ratings during the acquisition phase. (**B**) Show the negative emotion ratings during the acquisition phase. **: *p* < 0.01, ***: *p* < 0.001.

The ANOVA results for US expectancy ratings in the acquisition phase showed a significant main effect of stimulus type (*F*(1.66, 86.10) = 27.37, *p* < 0.001, η_p_^2^ = 0.35). Post-hoc tests (paired-samples *t*-tests) indicated that the US expectancy ratings were higher for the CS + negative (*M* = 6.40, *SD* = 1.18) than the CS− (*M* = 4.75, *SD* = 1.39, *t*(53) = 6.08, *p* < 0.001, Cohen’s *d* = 0.83), and the US expectancy ratings were higher for the CS + positive (*M* = 6.39, *SD* = 1.29) than the CS− (*M* = 4.75, *SD* = 1.39, *t*(53) = 5.60, *p* < 0.001, Cohen’s *d* = 0.76 (Fig. 4A). Other main effects and interaction effects were not significant (*ps* > 0.05). The results of the post-hoc tests indicated that participants successfully acquired the association between CS and US, and the learning degree for positive and negative social evaluations was not different.

The ANOVA results for negative emotion ratings in the acquisition phase revealed a significant main effect of stimulus type, *F*(1.94, 101.09) = 8.11, *p* = 0.001, η_p_^2^ = 0.14. Post-hoc tests (paired-samples *t*-tests) showed that the negative emotion ratings were higher for the CS + negative (*M* = 4.46, *SD* = 2.25) than the CS + positive (*M* = 3.13, *SD* = 2.05) (*t*(53) = 3.54, *p* = 0.001, Cohen’s *d* = 0.48 and the CS− (*M* = 3.39, *SD* = 1.89) (*t*(53) = 3.06, *p* = 0.003, Cohen’s *d* = 0.42) (Fig. 4B). The results of the post-hoc tests indicated that participants primarily responded with negative emotions to negative evaluations. Other main effects and interaction effects were not significant (*ps* > 0.05).

#### Generalization phase

##### Ratings results

The ANOVA results for US expectancy ratings in the generalization phase revealed a significant main effect of stimulus type, *F*(1.84, 95.41) = 56.03, *p* < 0.001, η_p_^2^ = 0.52. Compared to the CS− (*M* = 3.17, *SD* = 1.60), the US expectancy ratings were higher for the CS+ (*M* = 5.56, *SD* = 1.74), GS4 (*M* = 5.31, *SD* = 1.65), GS3 (*M* = 4.48, *SD* = 1.75), and GS2 (*M* = 3.55, *SD* = 1.64, *ps* < 0.001). No significant difference in US expectancy ratings was found between the CS + and GS4 (*p* = 0.87), and the GS1 and CS− (*p* = 1.00). Other main effects and interaction effects were not significant (*p* > 0.05). In terms of US expectancy, individuals’ evaluation generalization was mainly observed in the GS4, GS3, and GS2, and no group differences in generalization were found in the degree of generalization.

The ANOVA results for negative emotion ratings in the generalization phase showed a significant main effect of stimulus type, *F*(3.45, 179.12) = 2.67, *p* = 0.04, η_p_^2^ = 0.05. Participants’ negative emotion ratings were higher for the CS+ (*M* = 3.09, *SD* = 2.13) than the GS3 (*M* = 2.55, *SD* = 1.74, *p* = 0.02). The main effect of evaluation type was significant, *F*(1, 52) = 14.93, *p* < 0.001, η_p_^2^ = 0.22. Participants’ negative emotion ratings were higher for negative evaluations (*M* = 2.96, *SD* = 1.95) than positive evaluations. (*M* = 2.38, *SD* = 1.62, *p* < 0.001). The main effect of group was significant, *F*(1, 52) = 15.62, *p* < 0.001, η_p_^2^ = 0.23. Compared to the low SA group (*M* = 1.94, *SD* = 1.52), the high SA group (*M* = 3.23, *SD* = 1.80) reported higher negative emotion ratings (*p* < 0.001). The interaction between stimulus type and evaluation type was significant, *F*(3.58, 186.14) = 7.98, *p* < 0.001, η_p_^2^ = 0.13. Simple effects analysis revealed that participants provided higher negative emotion ratings for negative evaluations than for positive evaluations at the CS+ (CS + negative (*M* = 3.91, *SD* = 2.29) vs. CS + positive (*M* = 2.28, *SD* = 1.58) and the GS4 levels (GS4 negative (*M* = 3.26, *SD* = 1.93) vs. GS4 positive (*M* = 2.13, *SD* = 1.54) with *ps* < 0.001.

**Fig. 5:**
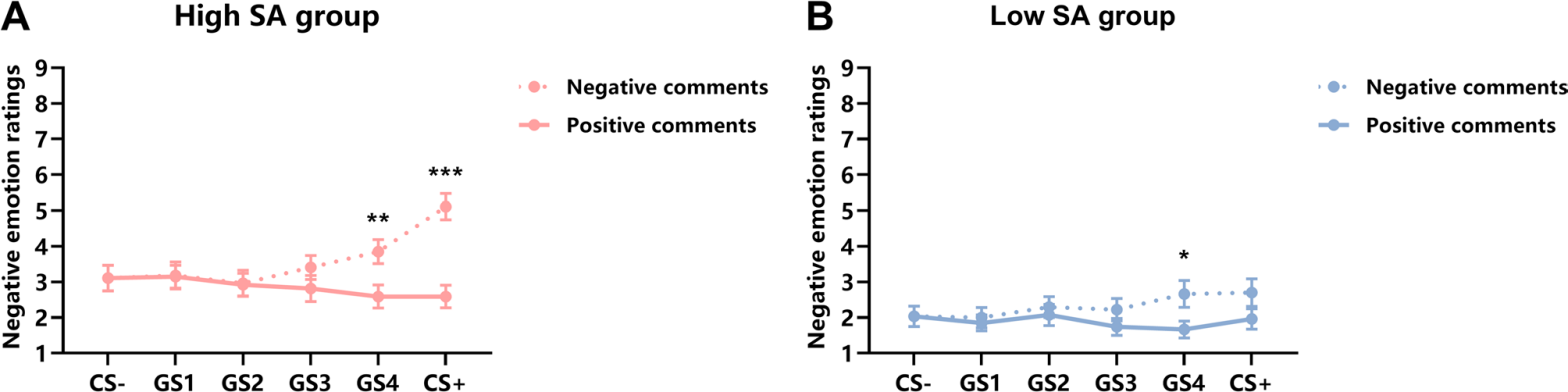
Negative emotion ratings results for high and low SA groups across the generalization phase in Experiment 2 (*M* ± *SE*). (A) show the negative emotion ratings during the generalization phase in high SA group. (B) show the negative emotion ratings during the generalization phase in low SA group. *: *p* < 0.05, **: *p* < 0.01, ***: *p* < 0.001.

The three-way interaction among stimulus type, evaluation type, and group was significant, *F*(3.58, 186.14) = 2.48, *p* = 0.05, η_p_^2^ = 0.05. Under the negative evaluation condition, the high SA group’s negative emotion ratings were higher for the CS+ (*M* = 5.11, *SD* = 1.91) than for the GS4 (*M* = 3.85, *SD* = 1.75) with *p* = 0.001, whereas the low SA group’s negative emotion ratings showed no significant differences (*ps* > 0.05). Under the positive evaluation condition, both groups’ negative emotion ratings showed no significant differences (*ps* > 0.05). The results indicated that individuals with high social anxiety were more sensitive to negative evaluations and exhibited stronger social evaluation generalization effects. Moreover, the high SA group’s negative emotion ratings for negative evaluations were significantly higher than ratings for positive evaluations at the CS + levels(CS + positive (*M* = 2.59, *SD* = 1.65) vs. CS + negative (*M* = 5.11, *SD* = 1.91) and GS4 levels (GS4 positive (*M* = 2.59, *SD* = 1.69) vs. GS4 negative (*M* = 3.85, *SD* = 1.75)) with *ps* < 0.004), while negative emotion ratings were significantly higher for negative evaluations than positive evaluations only at the GS4 level (GS4 negative (*M* = 2.67, *SD* = 1.94) vs. GS4 positive (*M* = 1.67, *SD* = 1.24, *p* = 0.02), with no significant differences in negative emotion ratings for the CS+, GS3, GS2, GS1, and CS− (*ps* > 0.05) (Fig. 5).

##### Slope results

**Fig. 6:**
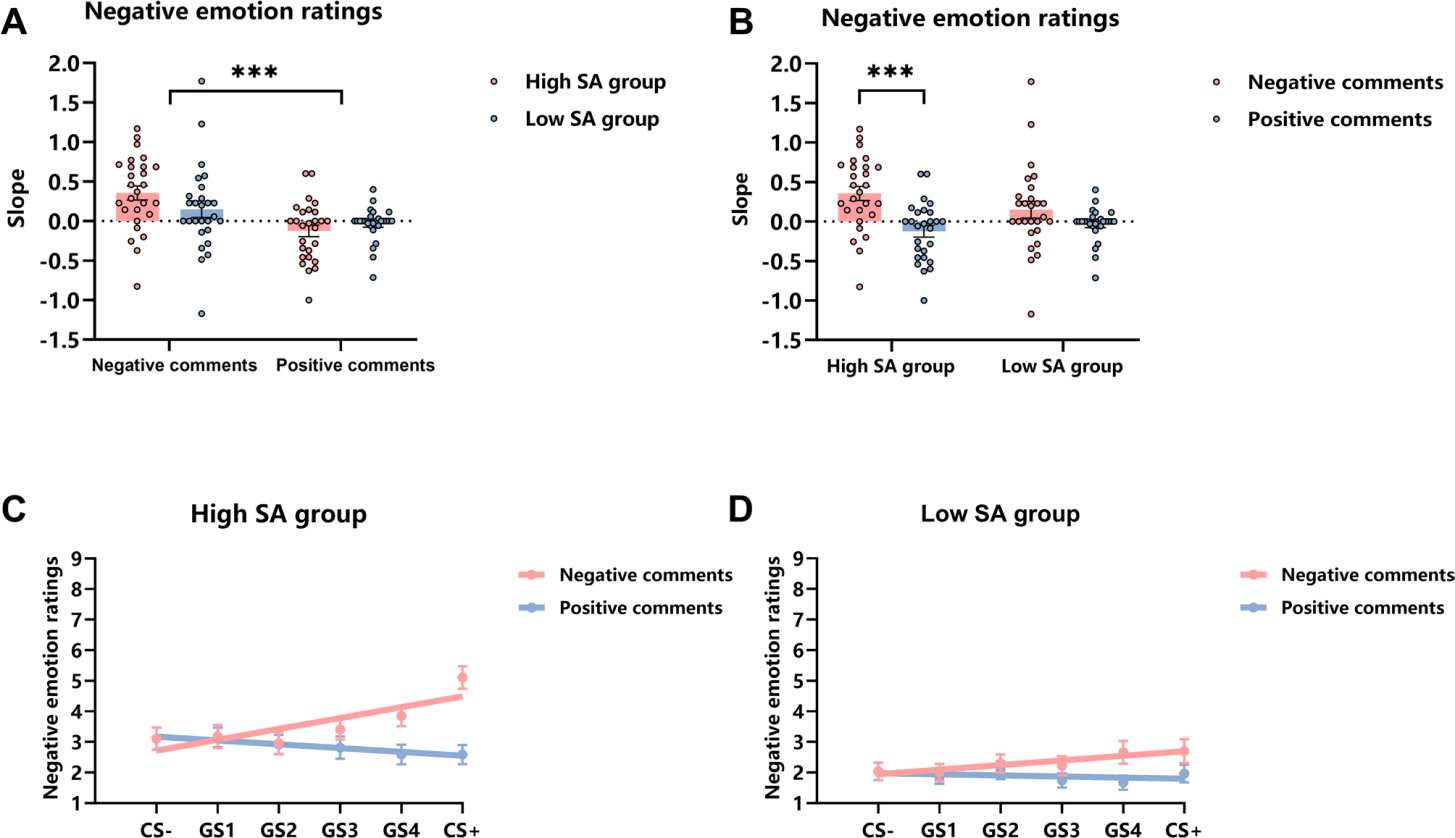
Results of linear slopes in the generalization phase of Experiment 2 (*M* ± *SE*). (**A**,**B**) Results of linear slopes for negative emotion ratings; (**C**,**D**) Linear slope fitting graphs for negative emotion ratings. ***p < 0.001.

The slope analysis results were mainly reflected in the negative emotion ratings. An ANOVA was conducted on the slopes calculated based on the negative emotion ratings. The results revealed a significant main effect of evaluation type, *F*(1, 52) = 20.86, *p* < 0.001, η_p_^2^ = 0.29. Post-hoc tests (paired-samples *t*-tests) showed that the slope was higher under the negative evaluation condition (*M* = 0.25, *SD* = 0.51) than under the positive evaluation condition (*M* = − 0.08, *SD* = 0.31), *t*(53) = 4.44, *p* < 0.001, Cohen’s *d* = 0.60 (Fig. 6A), indicating that participants were more sensitive to negative cues compared to positive cues. The interaction between evaluation type and group was significant, *F*(1, 52) = 4.06, *p* = 0.05, η_p_^2^ = 0.07. Simple effects analysis showed that the high SA group’s slope was stronger for negative evaluations (*M* = 0.36, *SD* = 0.47) than for positive evaluations (*M* = − 0.12, *SD* = 0.37, *p* < 0.001), whereas the low SA group showed no significant difference (*p* = 0.08) (Fig. 6B), further demonstrating that individuals with high social anxiety are sensitive to negative evaluations.

### Discussion

Building on Experiment 1, Experiment 2 further explored the differences in positive and negative social evaluation generalization among individuals with different levels of social anxiety. The results showed that participants successfully acquired the CS-US association between conditioned stimuli and evaluations, but exhibited higher levels of negative emotions towards negative evaluations, indicating more sensitivity to negative evaluations than to positive evaluations in a social context. In the generalization task, participants also demonstrated generalization effects in expectancy ratings and negative emotion ratings for GS, with the high SA group showing high overall levels of negative emotions and sensitivity to negative evaluations, the CS + and highly similar GS negative evaluation conditions, participants’ social evaluation generalization was more pronounced than in positive evaluation conditions. Slope analysis further demonstrated that high SA individuals were more sensitive to negative evaluations than positive evaluations, suggesting that individual differences might influence the generalization effect through hypervigilance and amplified interpretation of negative cues.

## General discussion

This study investigated how social anxiety levels influence the conditioned generalization of social evaluations through complementary web-based and lab-based paradigms. Both experiments provided convergent evidence for the relationship between social anxiety and evaluation processing: Experiment 1 demonstrated a significant positive correlation between social anxiety levels and negative evaluation generalization in expectancy ratings, whereas Experiment 2 showed that the high SA group exhibited enhanced generalization effects specifically in negative emotion ratings for negative evaluations. This differential sensitivity to negative evaluations manifested as heightened expectancy in Experiment 1 and intensified emotional responses to both CS + and similar generalization stimuli in Experiment 2. These findings align with the cognitive processing model of social anxiety, demonstrating how both expectancy bias and emotional reactivity to negative evaluations may contribute to the maintenance of social anxiety symptoms.

In the acquisition phase, US expectancy ratings confirmed successful CS-US learning across both experiments. The learning patterns were comparable for positive and negative evaluations, with higher US expectancy ratings for both CS + conditions compared to both CS − conditions. No significant differences were observed in the acquisition degree between positive and negative evaluations, indicating that participants effectively learned the contingencies regardless of evaluation valence. This equivalent initial learning provides an important baseline for interpreting the subsequent generalization effects, particularly the differential patterns observed for negative evaluations in individuals with higher social anxiety.

In the generalization phase, different patterns emerged for positive and negative evaluation generalization. For stimuli similar to the CS+, US expectancy ratings were significantly higher for negative evaluations compared to positive evaluations, a finding that was particularly evident in the lab-based experiment. This observation suggests an asymmetric processing of evaluation types, where enhanced expectancy for stimuli similar to the CS + indicates selective sensitivity in social evaluation processing. The analysis of negative emotion ratings revealed heightened responses in the high SA group, particularly under negative evaluation conditions. This enhanced emotional reactivity was evident not only for the CS+, but also extended to the most similar generalization stimulus (GS4). This broader generalization pattern aligns with the findings of Ahrens et al. (2016)^8^, who reported that negative emotional responses in socially anxious individuals extend beyond direct threat stimuli to perceptually similar stimuli. Such overgeneralization represents a key feature of anxiety disorders, as highlighted by Dymond et al. (2015)^6^. The results in the current study showed that positive evaluations reduced negative responses to stimuli similar to the CS−, indicating their protective role in social situations, a finding consistent with Carl, Gallagher, & Barlow (2018)^36^. Supporting these behavioral findings, the correlation analysis revealed a positive relationship between LSAS fear scores and the linear slope of negative emotion ratings specifically for negative evaluations. This pattern was particularly pronounced in the high SA group, which demonstrated significantly steeper slopes under negative evaluation conditions compared to positive evaluations, while the low SA group showed no such differentiation. These results align with Günther et al.‘s (2021) observation that socially anxious individuals exhibit selective attention to and enhanced processing of negative information, demonstrating how both expectancy bias and emotional reactivity contribute to the maintenance of social anxiety symptoms^37^.

The differential processing of negative versus positive evaluations observed in our study can be explained through evolutionary and psychological mechanisms. Dunsmoor (2015) suggested that negative information is very important for survival, resulting in enhanced vigilance and emotional reactivity to negative evaluations^38^. This evolutionary perspective helps explain why our high SA participants showed particularly strong generalization effects for negative evaluations. Importantly, our correlation analyses revealed that these effects were specifically linked to social fear rather than social avoidance. This pattern is consistent with the Clark and Wells (1995) model, which suggests that fear represents an immediate emotional response to social threats, while avoidance emerges as a secondary coping strategy^39^. This distinction helps explain why our observed effects were most pronounced in immediate emotional reactions to negative social evaluations.

Our findings bridge cognitive and learning perspectives in social anxiety through two complementary theoretical frameworks. The cognitive model of social anxiety^39^ predicts enhanced processing of social threats through biased attention and interpretation processes. Our results align with this model, demonstrating that high SA individuals showed enhanced generalization specifically for negative evaluations in both expectancy (Experiment 1) and emotional responses (Experiment 2). This enhanced processing reflects established patterns of attentional bias^40^ and interpretation bias^41^ in social anxiety. Classical conditioning principles^42^ further explain the mechanistic basis of these biases - while all participants showed basic generalization patterns following theoretical predictions about threat generalization^38^, the high SA group demonstrated selective enhancement for negative evaluations, suggesting that social anxiety alters basic learning mechanisms specifically for threat-relevant social information^43^. This integration of cognitive and learning perspectives^44^ demonstrates how cognitive biases may emerge through modified learning processes, with altered learning mechanisms serving as a pathway through which social anxiety symptoms are developed and maintained^45^.

The consistency between our Web-based and Lab results broadly supports the reliability of online experimentation in psychological research^15^. While both settings demonstrated fear generalization effects that positively predicted social anxiety, a central observation is the divergence in outcomes across these paradigms. The results from Experiment 1 indicated a link between social anxiety and biased cognitive expectancy, as reflected in US expectancy ratings, whereas the effect was primarily evident in affective ratings, specifically the negative emotion ratings in Experiment 2. We propose this divergence reflects a key functional dissociation between effortful cognitive biases and more automatic emotional biases, modulated by the availability of cognitive resources. Several interconnected factors likely contributed to this dissociation. Firstly, the web-based environment’s increased anonymity and the absence of an observing experimenter may have influenced participants’ responses to social evaluation. Specifically, the lack of a direct social presence could have reduced social desirability concerns, leading participants to report their internal, automatic emotional reactions more candidly. Secondly, from a cognitive perspective, other factors in the online experiment could have taxed cognitive resources for individuals with high social anxiety. These factors include the inherent noise of an online environment^46^ and a task framework that prompted self-monitoring through retrospective emotional ratings^47,48^. These results suggest that cognitive expectancies, being more dependent on controlled processing, were impaired by this combination of social and cognitive loads online. In contrast, affective ratings, which may be more automatic^49^, remained robustly expressed. We must emphasize that this framework is post-hoc; its primary value lies in generating a clear, falsifiable agenda for future research. The foremost task is, therefore, to test the causal role of cognitive resources, for instance by contrasting emotionally neutral ‘cold’ with socially-evaluative ‘hot’ cognitive loads, and to validate its external validity in contexts like Ecological Momentary Assessment (EMA) or Virtual Reality (VR). This work is crucial for understanding the complex expression of social anxiety and for developing more precise clinical interventions.

The present study provides initial evidence for the phenomenon of evaluative generalization, yet its correlational design and reliance on self-report represent notable limitations. The primary task for future research, therefore, is to move from correlation to causation. An experiment using the Trier Social Stress Test (TSST)^50^could directly test the causal role of negative affect by manipulating it experimentally and incorporating objective indicators, such as behavioral tasks. Building on this causal foundation, a deeper question concerns the underlying neural mechanisms. We propose that evaluative generalization stems from hyperactivity in threat-detection regions (amygdala/insula) coupled with hypoactivity in emotion-regulation regions (ventromedial prefrontal cortex, vmPFC). Given that a similar neural pattern has been identified in socially anxious individuals processing negative self-beliefs^51^, testing whether this mechanism extends to evaluative generalization using functional magnetic resonance imaging (fMRI) would be a critical next step.

Complementing the investigation of these core causal and neural mechanisms, future research should broaden its scope to the crucial question of who exhibits this bias more strongly and when. From a developmental perspective, a prospective longitudinal study focusing on adolescents, a peak period for social anxiety^52^, could examine whether early generalization biases predict the developmental trajectory of future social anxiety symptoms. From a cultural perspective, drawing on self-construal theory^53^, we hypothesize that negative social evaluation may elicit a greater self-threat, leading to more pronounced evaluative generalization in East Asian collectivistic cultures, which emphasize social harmony and the “interdependent self,” than in Western cultures. Finally, from a contemporary situational perspective, we propose that online social environments may exacerbate this bias by depleting the cognitive resources for emotion regulation via chronic cognitive load (e.g., persistent social comparison). This hypothesis can be tested directly in an experiment where participants browse social media before a task^54^, which we predict would transiently increase their generalization of negative evaluation.

## Summary

This study examined cognitive processing biases in social anxiety during social evaluation acquisition and generalization, and explored their associations with symptom levels. The key findings were (1) Anxiety was positively correlated with negative evaluation generalization, (2) Socially anxious participants showed greater generalization for negative evaluations than positive evaluations, and (3) Generalization effects were observed in both expectancy and emotion ratings, with the high social anxiety group showing greater sensitivity to negative evaluation cues. In contrast, these findings provide new insights into the cognitive mechanisms of social anxiety and potential clinical interventions. This study’s core contribution is not merely demonstrating the existence of evaluative generalization, but revealing a critical asymmetry within it: the generalization of negative social evaluation is far more potent than that of positive evaluation in social anxiety. This finding is important because it provides a concrete, mechanistic bridge between cognitive models and learning theories of social anxiety. It demonstrates that the power of negative social experiences to maintain and exacerbate anxiety lies not just in their occurrence, but in their disproportionate tendency to be psychologically amplified and generalized to new, safe contexts. Therefore, understanding and targeting this biased generalization process is not just a theoretical exercise, but a crucial step toward developing more precise and effective interventions for those suffering from social anxiety.

## Supplementary Information

Below is the link to the electronic supplementary material.

Supplementary Material 1

## Data Availability

All data can be found on our OSF repository (https://osf.io/q7hr2/).
