# Supplementary material for: Conditioned generalization of social anxiety effects based on positive and negative social evaluations
Source: Sci Rep. 2025 Dec 3;15:44168. doi: 10.1038/s41598-025-27857-2 (PMC12717244; doi:10.1038/s41598-025-27857-2)
Supplement: Supplementary file 1 — Supplementary Material 1 [file 41598_2025_27857_MOESM1_ESM.docx]

**Supplementary Materials**


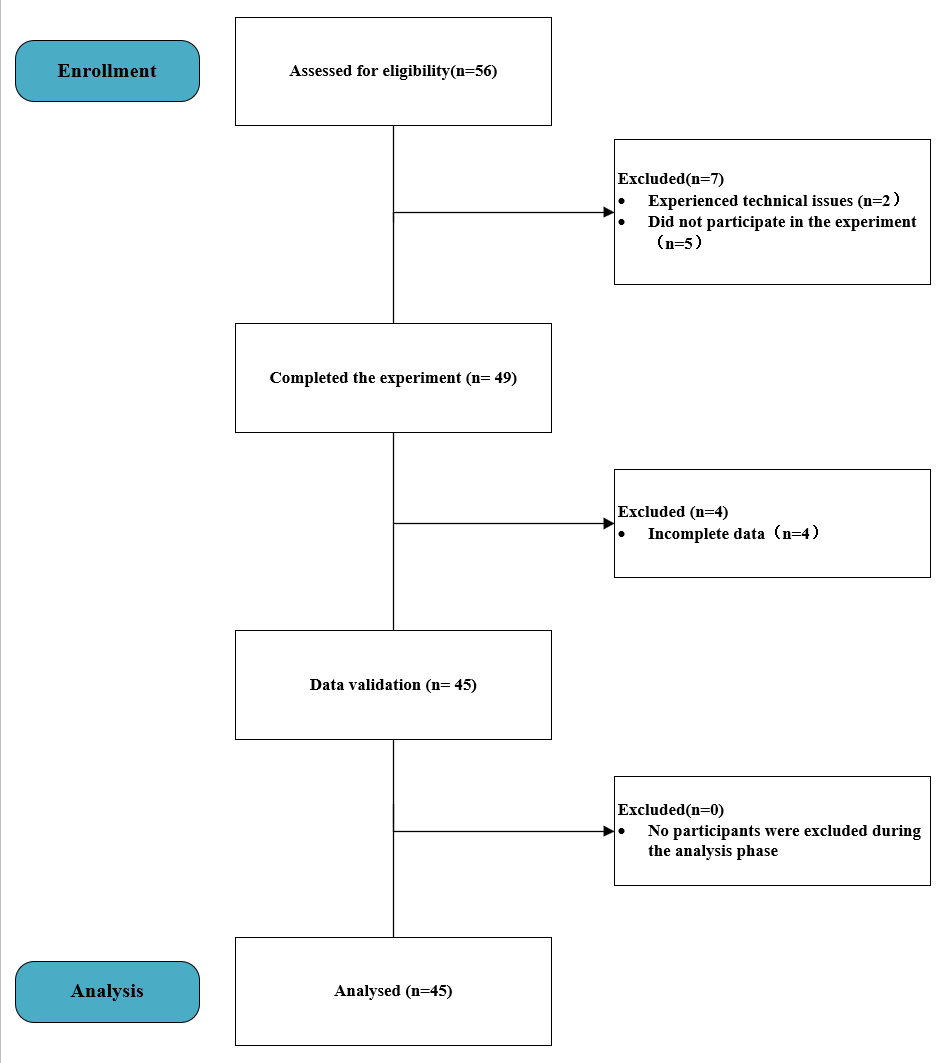


**Supplementary Fig.1:** In Experiment 1, among the 56 participants who underwent eligibility screening, 7 were excluded during the experimental phase: 2 due to technical issues with equipment and 5 who did not participate in the experiment. A total of 49 participants completed the experiment. During data validation, 4 participants were excluded due to incomplete data. No participants were excluded during the analysis phase. The final sample included 45 participants (34 females, mean age = 20.51 ± 1.80) who were included in the data analysis.


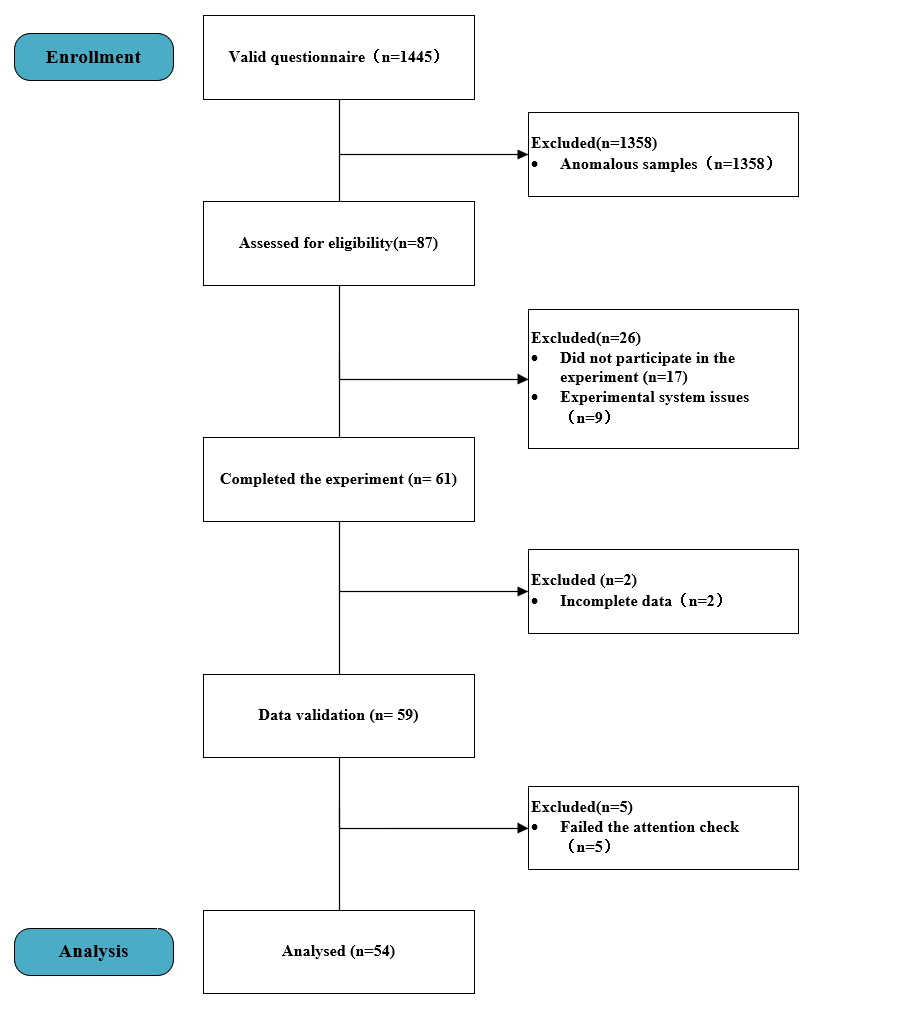


**Supplementary Fig.2:** In Experiment 2, for the series of studies, a participant pool was established through the Naodao Platform and social media, collecting 1,445 valid responses (78% valid response rate) from a total of 1,358 social anxiety questionnaires distributed. For this specific sub-study, to ensure distinct group differences in social anxiety levels, we specifically recruited participants from the upper and lower 20% of the LSAS score distribution. During the recruitment phase, 87 participants underwent eligibility assessment. During the experimental phase, 26 participants were excluded: 17 who did not participate in the experiment and 9 due to experimental system issues. A total of 61 participants completed the experiment. During completed the experiment, 2 participants were excluded due to incomplete data. In the subsequent analysis phase, 5 additional participants were excluded for failing attention checks. The final sample included 54 participants, who met all technical requirements for online participation (desktop or laptop computer with stable internet connection, quiet environment). The participants were divided into high SA group (27 participants; 13 females, mean age = 21.56 ± 1.99) and low SA group (27 participants; 14 females, mean age = 22.04 ± 3.25).
